# Supplementary material for: Are carbon emissions trading and green financial instruments synergistic? -Comprehensive quantitative research based on content analysis
Source: PLoS One. 2024 Mar 7;19(3):e0298601. doi: 10.1371/journal.pone.0298601 (PMC10919601; doi:10.1371/journal.pone.0298601)
Supplement: S1 Data — (ZIP) [file pone.0298601.s001.zip › Data/地级市Z_F工作报告.zip!/╨┬╜«/╬┌┬│─╛╞δ╩╨/└┤╫╘╣1⁄2╓┌║┼í╛┬φ┐╦╩2╛▌═°í┐_2016.docx]

各位代表：

现在，我代表市人民政府，向大会报告政府工作，请予审议，并请各位政协委员、列席代表提出意见。

“十二五”回顾和2015年的工作

“十二五”时期是首府经济发展最快、城乡面貌变化最大、各族群众得实惠最多的时期，也是全市社会稳定基础全面巩固、各项事业不断进步、城市地位和影响力显著提升的时期。在自治区党委、自治区人民政府和市委的坚强正确领导下，在市人大、市政协的监督支持下，我们紧紧围绕社会稳定和长治久安总目标，全面贯彻党的十八大和两次中央新疆工作座谈会精神，认真落实自治区党委、自治区人民政府和市委的重大决策部署，全力维护社会稳定，全力推动科学跨越，全力打造新疆首善之城，圆满完成了“十二五”目标任务，经济社会发展取得了巨大成就。特别是率先完成脱贫攻坚任务，全面建成小康社会总体指标实现程度达到97%，并有十个方面走在全疆全国前列：经济保持持续高速增长，主要经济指标增速始终位于全国省会（首府）城市前列。基层基础空前加强，走出了一条具有首府特色的基层组织建设之路。创造性实施“煤改气”工程，大气污染得到根本性治理。综合交通枢纽建设实现大跨越，创造了西部地区交通建设发展的奇迹。成功创建国家园林城市，获得全国文明城市提名资格，通过国家双拥模范城“八连冠”检查验收。棚户区、老旧小区改造和农村“两居”工程建设规模空前，各族群众居住条件显著改善。城市道路清扫保洁率达100%，冬季清雪做到“即下即清、雪停路净”，均达到国内领先水平。建成覆盖全市的527个社区蔬菜副食品直销点，稳价惠民。实行“区政合一”，两个开发区财政收入分别突破百亿元大关。创造性开展“群众满意好班子”争创活动，干部队伍执政为民能力显著提升。

——稳定的基层基础全面巩固。五年来，面对复杂的反恐维稳形势，我们牢固树立“五个观点”和“五个宁可”思想，坚持打团伙、查“三非”、加强流动人口和出租房屋管理三项重点工作，切实打牢基层组织基础、维稳力量基础和群众工作基础。深入开展民族团结教育，依法加强对宗教事务的管理。

——经济实现跨越式发展。五年来，我们坚持稳中求进、改革创新，加快构建现代产业体系，着力推动经济转型跨越发展，预计2015年主要经济指标均比2010年翻一番多。其中，地区生产总值由2010年的1338亿元增加到2015年的2680亿元，增长1倍；固定资产投资由483亿元增加到1708亿元，增长2.5倍；社会消费品零售总额由563亿元增加到1152亿元，增长1.04倍；全口径外贸进出口总额由64.2亿美元增加到136亿美元，增长1.12倍；全口径财政收入由433亿元增加到1040亿元，增长1.4倍；地方财政收入由197亿元增加到465.1亿元，增长1.36倍。“五大产业基地”初具规模，经济总量和财政收入已占全市50%左右。金融、物流、旅游、会展和总部经济快速发展。

——城市面貌发生历史性巨变。五年来，我们坚持规划先行和一切按规划办事，大力加强城乡规划建设管理，城市环境和品质进一步优化提升。累计筹措2000多亿元用于城市建设，是“十一五”时期的7.3倍。实施了“两线一绕”、“两桥一路”等一大批道路交通、供排水和环保生态重点项目，“田”字路、兰新高铁等重大交通工程相继建成，地铁、城际铁路、国际机场四期改扩建等重大项目加快推进，综合交通枢纽日臻完善。着力构建多中心、组团式城市发展格局，高铁、会展、白鸟湖新区形成规模，五年拓展城市空间200多平方公里。持续开展大规模环境综合整治，深入推进以园林绿化为重点的生态环境建设，新建小游园小水面及街旁绿地737块，新增绿化面积25.3万亩，城市绿化覆盖率达40.3%。超额完成4项污染物总量减排任务，减少燃煤消耗1500万吨，万元GDP能耗大幅下降。

——改革开放取得重大突破。五年来，我们坚持深化改革、扩大开放，开创了首府经济社会发展的新局面。建立中心城区“大政法委”和“大建委”体制，在大部分复杂区域实行管委会建制，推进社区实体化建设，构建了社会管理新格局。调整城市管理和社会服务部门设置，实行规划土地市级统一管理。推进管理重心下移，向区（县）下放509项市级建管权限。推进行政审批制度改革，压减审批事项50%以上。兵地融合发展稳步推进。对外合作交流不断扩大，累计实施招商引资项目1946个，引进区外到位资金2491亿元；实际利用外资11.15亿美元；境外投资企业达127家。成功举行四届中国—亚欧博览会，举办三届丝绸之路经济带城市合作发展论坛，“五大中心”建设全面启动，缔结友好城市总数达27个，我市成为丝绸之路经济带建设中倍受瞩目的城市。

——民生改善取得显著成效。五年来，我们持续开展“民生改善年”活动，市财政用于民生领域支出达1236亿元，占同期一般公共预算支出的71.4%，较“十一五”时期增长3.2倍。累计投入454亿元，实施了以“煤改气”工程为重点的126个大气污染治理项目，空气质量达标天数在全国74个重点监控城市中由排名末位上升到前20位左右。投入350多亿元，建设棚改房、保障房和农村“两居”房18万余套，惠及80余万群众。公共交通体系不断完善，建成国内一流的BRT系统7条线101.69公里，入选国家“公交都市”示范城市首批创建城市。新增城镇就业近46万人，城镇登记失业率控制在3.7%以内，城镇职工、居民养老医疗等社会保障水平不断提高。城镇居民人均可支配收入和农牧民人均纯收入分别由14402元和7471元增加到31500元和15200元，增长1.19倍和1.03倍。教育经费年均增长22.76%，各类教育教学水平稳居全疆首位。公共卫生和医疗服务体系进一步完善，基本公共卫生服务均等化水平显著提高。安全生产基础不断夯实，监管能力进一步增强。公共文化服务体系不断健全，文化惠民活动扎实开展，城乡广播电视覆盖率达到99.8%，累计放映公益电影4.43万场，群众精神文化生活日益丰富。每年向社会庄严承诺的百件民生实事全部如期完成，受到各族群众的真情赞誉！

2015年是“十二五”收官之年，也是自治区成立60周年。一年来，我们认真贯彻落实市委十届十一次全委（扩大）会议精神，圆满完成了市十五届人大四次会议确定的目标任务，为各族群众献上了满意的答卷。

一、社会大局持续稳定

严打严防措施不断强化。持续开展严打专项行动，及时打掉一批危安团伙，为社会稳定消除了重大安全隐患。持续巩固基层维稳基础，扎实开展“访惠聚”活动。“去极端化”工作深入推进。认真落实自治区“去极端化”工作部署，进一步加强“三非”治理，有效遏制了宗教极端思想传播。民族宗教工作扎实开展。大力开展党的民族理论、民族基本知识和马克思主义“五观”、“三个离不开”、“五个认同”宣传教育。市、区（县）“两模范”、“双五好”创建申报率均超过50%，命名表彰民族团结模范社区（村）381个、民族团结模范单位801个，“五好”宗教活动场所250个，“五好”宗教人士245名。组织宗教人士投身文明城市创建和公益慈善事业，积极引导宗教与社会主义社会相适应。

二、经济保持平稳较快发展

预计全年地区生产总值增长10.5%；社会固定资产投资增长12%；社会消费品零售总额增长7.6%；全口径外贸进出口总额增长3%；地方财政收入增长2.7%，其中一般公共财政预算收入增长8.2%；城镇居民人均可支配收入增长17.1%；农牧民人均纯收入增长14.2%。

积极应对经济下行压力。制定应对经济下行26个方面70条措施，全力化解经济运行中的困难和问题。落实各项减税和“营改增”政策，为纳税人总体减负62亿元，增强了企业信心，激发了经济发展活力。抓好284个市级重点项目建设，累计完成投资910.35亿元。积极争取中央和自治区各类支持资金，置换政府债券资金168亿元；与中交建、中铁建、中铁工、中建集团签署轨道交通、轻轨、中低速磁悬浮铁路、城南经贸合作基地等基础设施建设战略合作协议，总金额超过5000亿元，为增强首府发展后劲奠定了坚实基础。

“六大产业基地”规模不断扩大。先进装备制造业基地、高新技术产业基地和战略性新兴产业基地集聚引领作用日益增强，工业增加值占到全市工业增加值的30%左右。城南经贸合作基地基础设施建设先行启动，11条道路全面开工。批准实施南山旅游基地总体规划，天山大峡谷景区游客中心建成运营。“六大产业基地”产业规模和综合实力快速提升，已成为推动首府经济结构调整、转变发展方式、促进经济增长的新引擎。

科技创新能力持续增强。新增自治区级企业技术中心4家、产学研联合开发示范基地11家、市级工程技术研究中心及重点实验室6家，高新技术企业达216家、创新型（试点）企业达128家。创新开展“银政通”融资业务，搭建科技金融战略联盟，为308家企业融资22.78亿元。举办首届乌鲁木齐创新创业大赛，24个创新创业团队获得自治区及我市奖励。专利申请量达4700件，增长15%；专利授权量3800件，增长80%。

新型工业化快速推进。实现工业增加值625亿元，完成工业投资435亿元。新能源装备制造、电力行业快速发展，非石油工业占工业的比重达62%。中国铁建重工、南车株洲电力机车落户我市，填补了全疆地下工程及轨道交通高端装备制造产业空白。达坂城风电场二期等12个重点项目建成投产。淘汰工业过剩产能95万吨，万元工业增加值能耗下降10.8%；关闭煤矿25家，淘汰产能231万吨。第四代移动通信基础设施建设完工，智慧城市、云计算产业基地曙光云项目快速推进。

服务业升级步伐加快。实现服务业增加值1860亿元，增长13.9%。建成新疆软件园和三大数据库，“五大物流园”、乌鲁木齐国际纺织品服装商贸中心等项目加快推进，大型城市综合体项目进展顺利。经济技术开发区（头屯河区）获批国家电子商务示范基地，唯品会新疆运营总部成功落地。城市共同配送体系试点加快实施，肉类蔬菜流通追溯体系初步形成。建成特色餐饮街、美食城16个，国家级钻级酒家酒店100家。主办承办各类展会112个，荣获2015年度“中国十佳优秀会展城市”。接待国内外游客2203.4万人次，增长9.37%；旅游总收入298.35亿元，增长8.66%。丝绸之路经济带旅游集散中心、中国旅游产业创新服务基地建设全面启动，荣膺“美丽中国之旅十佳绿色生态城市”。

农业农村工作成效显著。统筹城乡发展，全面完成3.8万套“两居”房建设任务，改善了10万余名农牧民住房条件。农村相对贫困人口最低收入高于全国2020年脱贫线600元，达到4600元。实施压粮节水7.6万亩，新建温室和大棚6133亩、标准化养殖小区（场）50个，完成百万肉羊（牛）生产体系项目建设。新增农产品质量安全检（监）测点52个，检测合格率达97%。新建动物疫病监测点8个，建成重大动物疫病防控监管视频应急指挥平台。实施农村安全饮水项目26个，新建农村供水管线259.6公里、农村公路76公里。

三、民生改善力度持续加大

百件民生实事全面落实。取消河滩路、外环路车辆通行费，政府少收和承担26亿元。新建投运BRT4号线、6号线，开通和优化公交线路22条，新增和更新新能源公交车350辆，建设农村班线港湾式车站40座，启动智能交通指挥中心建设。扩大市级肉菜储备和投放，加强社区蔬菜副食品直销点运营监管，居民消费价格总水平涨幅为1%。投资45亿元，实施棚户区改造项目19个、建设房屋7180套；新建保障性住房1.2万套，3923户家庭圆了“安居梦”。制定加强住宅小区物业服务管理的意见，健全四级物业管理体系，在46个老旧小区开展专业化物业管理。建成市社会福利中心和104个社会老年活动中心、10个老人照料中心。

就业和社会保障体系不断健全。新增城镇就业10.01万人，开展各类职业技能培训11.98万人，农村富余劳动力转移就业6419人，创业就业1.8万人。实施离校未就业高校毕业生就业促进计划，实现高校毕业生就业1.39万人。完成市级人力资源市场和58个一站式就业社会保障服务大厅建设。创建充分就业社区47个，零就业家庭持续动态清零，城镇登记失业率控制在3.5%。建立统一的城乡居民基本养老保险制度，在全疆率先开展新农合与城镇居民基本医疗保险并轨工作。五项社会保险净增9万人。机关事业单位养老保险制度改革稳步推进。企业退休人员月均养老金待遇提高到2636元，城镇职工最低工资标准提高到每月1470元，城乡居民最低生活保障标准分别提高到每月380元和195元。

教育、卫生、体育等各项社会事业全面发展。投入70.42亿元，新（改扩）建中小学校32所，建成128个城乡学校少年宫和8个活动中心。区（县）义务教育学校标准化建设全面完成，5个区（县）义务教育均衡发展通过国家验收。设立普惠性公益性民办幼儿园发展奖补资金，新增公办幼儿园5所。落实“两免一补”等各类资金12.64亿元，惠及95.4万人次。职业教育办学效益明显，职业学校毕业生就业率达96%。加快医疗卫生基础设施建设，乌鲁木齐国际医院主体完工、友爱医院建设加快推进。全面深化医药卫生体制改革，4家县级公立医院实施药品零差率销售，社区卫生服务机构实现基本药物制度全覆盖，人均基本公共卫生服务经费补助标准稳居全疆首位。人口和计划生育服务管理不断加强，人口出生率为8.63‰。扎实开展食品药品专项整治，群众饮食用药安全得到保障。深入开展安全生产领域“打非治违”等专项行动，各类安全生产事故下降34.9%。高标准建成第十三届全国冬运会冰上运动场馆，完成红山体育馆改造工程，为142个社区和6个乡镇配置全民健身设施。圆满完成了全国高山滑雪锦标赛、十三届冬运会男子冰球资格赛等重大体育赛事服务保障工作。在全国第一届青年运动会上取得4金6银8铜的好成绩，获得全国校园足球总决赛初中和小学组冠军。新闻出版、防灾减灾、质监、气象、地震、人防、地方志、档案等工作取得新进展，妇女儿童、残疾人和红十字事业取得新成绩。

四、城市规划建设管理水平不断提升

城乡规划不断完善。编制完成“十三五”经济社会发展规划纲要和117个专项规划。城市总体规划修改工作扎实推进，中心城区控规覆盖率达到90%。编制了会展片区、高铁片区、二道桥片区、大湾片区等7项城市设计，完成了消防、综合防灾等27项专项规划。

基础设施建设快速推进。投入350亿元实施了183个城市基础设施和维护项目。绕城高速公路东线全线贯通，东二环征收工作全面完成。阿勒泰路高架等一批城市道路竣工通车，乌奎连接线、乌五公路改扩建工程顺利实施；高铁新客站站房建成，开通至吐鲁番、库尔勒的城际铁路。地铁1号线全面开工，启动地铁2号线一期工程建设。铁路集装箱中心站建设快速推进，公铁联运汽车站、三屯碑快速公交枢纽工程基本建成。完成“500”水库城北调水工程，新建供排水输配管网121公里，西山750千伏、南郊220千伏、水西沟110千伏输变电工程建成投运。实施大浦沟垃圾填埋厂治理工程，启动米东垃圾综合处理厂项目建设。

城市管理水平进一步提高。围绕迎接自治区成立60周年，高水平办好6项重大活动，高标准做好服务保障、环境整治、氛围营造等工作，圆满完成大庆任务。重点提升“10大片区”环境，打造示范街10条、达标路19条，粉刷楼体1106栋、修补道路106万平方米；拆除违法建筑112.9万平方米、户外广告设施6521块。持续推进城市管理重心下移，区（县）责任进一步落实。环卫精细化管理水平不断提高，主次道路基本实现湿法机械化清扫作业，生活垃圾无害化处理率达95.8%。

大气污染治理成果不断巩固。投入70亿元实施了14个大气污染重点治理项目。完成燃气锅炉烟气余热冷凝改造2220蒸吨，拆改燃煤小锅炉1789台，实施换热站温控节能改造95座，改造老旧供热管网282公里。开展四大电厂和企业自备电厂综合治理，完成12家重点企业污染源治理。关停并转米东区污染企业480家。淘汰“黄标车”及“老旧车”1.2万辆。实施既有建筑节能改造155万平方米，建成南部集中供热配套管网29.79公里。启动达坂城区和高铁片区电热采暖试点，开展高铁片区中德低碳生态示范城区建设，实施超低能耗建筑示范项目5.62万平方米。全年空气优良天数达313天，优良率达85.75%，首府环境空气质量持续改善。

生态建设成效明显。第十二届中国国际园林博览会申报和选址工作加快推进，国家环保模范城市顺利通过自治区初评，全国节水型城市通过复验。新增绿化面积3.5万亩，庭院绿化2500亩，新建小游园小水面及街旁绿地108块，启动东绕城生态圈和7个荒山绿化公园建设。创建“花园式”单位20个、花园社区5个，新增绿化合格单位25个。推进乌鲁木齐河流域生态保护，一号冰川、柴窝堡湖、盐湖等重点水源涵养区环境综合整治顺利实施。

五、改革开放全面深化

重点领域改革稳步推进。在全疆率先公布市级部门3275项行政权力清单，将行政许可事项减少到154项。发布政府核准投资项目目录，大力推广政府与社会资本合作（PPP）模式，推出43个PPP试点项目，总投资574亿元。深化商事制度改革，启用信用联合监管平台，注册登记实现“三证合一”、“一照一码”，新增市场主体4.7万个，增长14.8%。推进实施不动产统一登记。合作设立普惠民生发展基金、城市建设基金和红山创业投资基金，成功发行第一支创新型小微企业增信集合债券。制定深化国有企业改革实施意见，推进国有资产统一监管。合资成立南车轨道交通装备有限公司，引入战略投资者推动友好集团业务优化升级。

以丝绸之路经济带核心区“五大中心”建设为基础的对外开放格局初步建立。完成“五大中心”建设专项规划，启动实施了一批重大基础设施项目。成功举办第三届丝绸之路经济带城市合作发展论坛，成立了论坛理事会和秘书处。乌鲁木齐综合保税区成功获批并启动建设。积极申报乌鲁木齐自由贸易试验区，已获自治区支持并上报国务院。获批全国多式联运海关监管中心，建成各类外贸基地11个，跨境电子商务公共服务平台建设取得积极进展。开通中欧、中亚班列80列，实现常态化运行。完成区域医疗信息平台建设，成功举办丝绸之路健康论坛—健康产业高层研讨会、与周边国家健康合作研讨会，签署10个重要合作协议。完成招商引资项目305项，引进区外到位资金694亿元，增长11%；新批外商投资项目20个，实际利用外资1.94亿美元，增长11.9%。

六、现代文化建设取得新进展

围绕社会主义核心价值体系建设，广泛开展主题宣传教育，扎实推进公民道德建设和文明城市创建。开展学雷锋志愿服务和文明交通出行等主题活动，抓好文明礼仪、文明秩序、文明环境建设，强化服务行业和窗口单位文明服务创评。创建市级文明示范社区（村）56个，评选表彰道德模范32名、美德少年20名。市民对文明创建的知晓率、支持率、参与率、满意率明显提高，在中央文明委第一年度创城测评验收中受到好评。公共文化服务体系不断完善，市文化中心“六馆”项目加快推进，新建村文化室25个。文化惠民工程深入实施，组织开展各类群众文化活动3400场（次），政府购买文化惠民演出310场，放映公益电影9616场。文艺精品创作不断繁荣，创作了大型交响乐《保卫黄河》、大型歌舞《时代之歌》等一批新剧目，开展第二届“红山文艺奖”评选工作。参展第十一届深圳文博会，签约10个文化产业项目，金额达22.2亿元。

七、政府自身建设全面加强

巩固和深化党的群众路线教育实践活动成果，扎实开展“三严三实”专题教育，各级领导干部思想政治建设和作风建设明显改善。依法治市工作扎实推进，创建法治先进单位、社区（村）1217个，圆满完成“六五”普法总结验收。依法行政和法治政府建设稳步推进，提请人大常委会审议地方性法规草案1件，制定政府规章7件；自觉接受人大法律监督和政协民主监督，办理自治区、市人大代表议案、建议和政协提案548件，办复率100%。区、市两级政务服务中心（公共资源交易中心）主体工程封顶，政府购买服务力度进一步加大，服务型政府建设加快推进。政务公开和网络问政工作不断加强，市长专线与城市管理服务热线并线运行，受理市长专线14565件、市长信箱1707件，办结率100%；网络问政4454件，回复率98.7%。信访和行政调解工作机制进一步完善。行政监察、审计监督工作扎实推进，严肃查处了一批贪污受贿、损害群众利益的违法违纪案件，预防和惩治腐败取得新成效。

各位代表，“十二五”时期，我们在维护社会稳定和长治久安、全面建成小康社会的征程中，取得了令人瞩目的成就，这是自治区党委、自治区人民政府和市委坚强正确领导的结果，是市人大和市政协监督支持的结果，是全市上下团结奋斗的结果。在此，我代表市人民政府，向全市各族干部群众，向驻乌部队、武警指战员、公安干警、兵团第十二师职工和援疆援乌干部，向人大代表、政协委员、各民主党派人士，向所有关心支持首府稳定发展的各界朋友，表示衷心的感谢，并致以崇高的敬意！

在肯定成绩的同时，我们也清醒地看到，当前首府经济社会发展还存在一些问题和不足：一是反恐维稳仍需做大量深入细致的工作。二是经济下行压力依然较大，部分重点行业和企业经营效益持续下滑，转方式、调结构、稳增长任务十分艰巨。三是城市基础设施建设仍需加强，生态环境保护还需加大力度，大气污染区域联防联控难度较大，加快发展受土地、水等资源约束趋紧。四是公共服务体系还不健全，基本公共服务均等化水平有待提升，教育、医疗、就业和社会保障等方面条件还需进一步改善，科技创新进步、人才队伍建设、农业农村工作等方面仍存在不少问题短板。五是城市文化特色不强，精神文明建设仍需加大力度，市民文明素质和社会文明程度需要进一步提升。六是重点领域改革需要进一步突破，涉及稳定与发展的深层次矛盾问题依然较多。七是政府职能转变与形势任务要求还不相适应，服务群众能力还需进一步提高。对于这些问题和不足，我们将高度重视，采取有效措施切实加以解决。

“十三五”目标任务和2016年的主要工作

“十三五”时期，是首府维护社会稳定和实现长治久安的重要时期，是全面建成更高水平小康社会的决胜阶段，也是我们朝着建设现代化国际城市目标迈进的关键时期。我们将认真贯彻落实市委十届十四次全委（扩大）会议精神，推动首府经济社会在新的起点上，实现更长时间、更高水平、更好质量的发展。

“十三五”时期政府工作的指导思想是：高举中国特色社会主义伟大旗帜，以邓小平理论、“三个代表”重要思想、科学发展观为指导，深入贯彻习近平总书记系列重要讲话精神，紧紧围绕社会稳定和长治久安总目标，坚持“五位一体”总体布局和“四个全面”战略布局，坚持依法治疆、团结稳疆、长期建疆总体方略，坚持创新、协调、绿色、开放、共享发展理念，统筹推进经济、政治、文化、社会和生态文明建设，落实城市工作“尊重一个规律、坚持五个统筹”的要求，着力优化“四大环境”、建设“五大中心”、提升“六大产业”、构建“七大组团”，全面深化改革开放，全面推进依法治市，全面建成更高水平的小康社会，为把乌鲁木齐建设成为现代化国际城市和全疆团结和谐、繁荣富裕、文明进步、安居乐业的首善之城而努力奋斗。

“十三五”时期我市经济社会发展的主要目标是：国民经济保持中高速增长，地区生产总值年均增长7.5%以上，社会固定资产投资年均增长12%，社会消费品零售总额年均增长10%，一般公共财政预算收入年均增长7%，全口径外贸进出口总额年均增长7%，城乡居民人均收入年均增长9%以上，物价总水平保持基本稳定，科技研发投入大幅增加，万元GDP能耗明显下降；城市现代化国际化功能完备；人民生活水平和质量大幅提高；生态环境质量进一步改善；城市文明程度显著提升；民族团结宗教和谐明显加强；社会大局保持持续稳定。做好“十三五”各项工作，重点把握好以下六个方面：

一是坚决维护社会稳定，不断夯实社会稳定和长治久安基础。围绕社会稳定和长治久安总目标，坚决贯彻中央、自治区党委和市委的一系列重大决策部署，不断强化“五个观点”和“五个宁可”思想，加强民族团结、促进宗教和谐、“去极端化”、遏制宗教极端思想渗透蔓延为重点，进一步加强基层基础建设，全面落实维稳常态化措施，不断巩固社会稳定和长治久安的根基。进一步健全民主法治，推进依法治市，提高社会治理体系和治理能力现代化水平，建设平安首府和法治首府。

二是坚持创新发展，着力提高发展质量和效益。切实把发展基点放在创新上，深入实施创新驱动发展战略，建设丝绸之路经济带创新驱动发展试验区。培育发展新动力，着力加强从供给端推动产品品质提升，更好地满足群众日益升级的物质文化需求。构建产业发展新体系，突出先进装备制造、新能源、新材料、纺织服装、特色医药等工业领域，金融、物流、旅游、教育、医疗、信息、检验检测等服务领域，逐步形成优势明显、定位明确、具有国际竞争力的产业新体系。拓展城市发展新空间，按照“尊重一个规律、坚持五个统筹”的要求规划城市，建设和完善一批枢纽型、功能性、网络化的重大战略性基础设施，全面建成“七大组团”新区，促进产城融合。打造发展新优势，实施“互联网+”和“+互联网”行动，支持云计算、物联网等新一代信息技术研发和产业化，构建信息丝绸之路西向大通道国际性枢纽，基本建成智慧城市。营造创新发展新环境，构建众创空间，培育创客主体，推动大众创业、万众创新。深化科技等相关领域改革，强化创新发展的体制机制保障，建成国家创新型城市。

三是坚持协调发展，着力增强发展的整体性。坚持理念引领、问题引领和实践引领，加快转方式、调结构、补短板，巩固提升“六大产业基地”，不断增强产业竞争力。以现代服务业基地、南山旅游基地和城南经贸合作基地建设为重点，引导生产要素集聚发展；以先进装备制造业基地、高新技术产业基地和战略性新兴产业基地为重点，加快推进新型工业化，力争到2020年成为全疆新型工业化引领之地。促进城乡一体化发展，加快都市农业现代化，提高社会主义新农村建设水平。推进区域协调发展，全面落实天山北坡经济带发展规划，增强首府服务区域、服务全疆的功能，辐射带动周边区域加快发展。加快兵地深度融合发展步伐，提升兵地合作区发展水平。推动物质文明和精神文明协调发展，提升城市文明程度，成功创建全国文明城市。统筹推进公共文化服务均衡发展，实现文化产业大发展，基本建成西部文化大市。加强全民国防教育和后备力量建设，不断增进军政军民团结。

四是坚持绿色发展，着力建设天蓝地绿水清的美丽家园。牢固树立环保优先、生态立市理念，建立健全生态文明体制机制，更加注重源头治理和长效机制建设。完善大气污染联防联控体系，优化城市能源消费结构，加大工业污染源减排力度，强化扬尘污染和机动车尾气治理，持续改善环境空气质量。提升城市生态绿化水平，全面建成绕城生态圈防护绿带，构建首府绿色生态屏障。实施生态系统分区分级保护，推进重大生态修复工程。加大环境污染治理力度，推进全社会节能减排，扩大清洁能源应用与覆盖范围，促进绿色循环低碳发展，实现创建国家生态园林城市、卫生城市和环保模范城市目标。

五是坚持开放发展，着力提升开放型经济水平。紧紧抓住“一带一路”建设给首府发展带来的重大历史机遇，加快现代化国际城市和丝绸之路经济带核心区“五大中心”建设，推进从丝绸之路经济带节点城市向中心城市跨越。以中国—亚欧博览会、丝绸之路经济带城市合作发展论坛为重要平台，进一步深化同国内外城市多领域互利共赢的务实合作，着力拓展对外经贸交流合作新渠道，打造内外联动、东西双向开放的全面开放新格局。扎实做好乌鲁木齐自由贸易试验区申报工作，加快跨境电子商务示范城市、服务外包示范城市申报建设，推进外贸产业转型升级。

六是坚持共享发展，着力增进人民福祉。按照人人参与、人人尽力、人人享有的要求，着力形成持续改善民生的长效机制，使各族市民共享改革发展成果。增强公共产品与公共服务供给，提高公共服务共建能力和共享水平。加快社会事业改革发展，坚持教育优先发展，促进起点公平和机会公平。深化医药卫生体制改革，完善公共卫生服务体系，健全覆盖城乡居民的基本医疗卫生制度。坚持计划生育基本国策，促进人口均衡发展。实施更加积极的就业政策，鼓励以创业带动就业，推动实现更加充分、更高质量的就业。完善城乡统一的社会保障制度，兜住兜牢人民群众生活底线。进一步改善居住环境，力争到2020年基本完成棚户区改造任务。积极应对人口老龄化，完善养老服务体系，增强养老服务供给能力，不断提高各族群众生活质量和满意度。

2016年是“十三五”的开局之年，也是首府改革发展稳定至关重要的一年。我们将按照市委十届十四次全委（扩大）会议的部署要求，狠抓落实，扎实工作，努力实现“十三五”良好开局。今年全市经济社会发展的主要目标是：地区生产总值增长7%—7.5%，社会固定资产投资增长12%，社会消费品零售总额增长8%，全口径外贸进出口总额增长6%，一般公共财政预算收入增长5%，城镇居民人均可支配收入增长8%，农牧民人均纯收入增长9%，居民消费价格总水平涨幅控制在2%左右，城镇登记失业率控制在3.7%以内。围绕这一目标，我们将重点做好以下工作：

一、推进维稳工作常态化，筑牢社会稳定和长治久安根基

一是打防结合，堵塞漏洞，狠抓各项维稳措施落实。始终坚持主动进攻、先发制敌，不断深化严打斗争。始终坚持以防为先、立足于防，发挥人防物防技防作用。

二是加强基层基础建设。继续抓好以片区管委会（街办、乡镇）、社区（村）为重点的基层组织建设，完善片区管委会管理机制，巩固提升社区“十有”条件，让基层组织在维护稳定和服务群众中发挥更大作用。加强基层干部队伍建设，加大教育培训力度，注重实践锻炼培养，不断提高素质能力。扎实开展第三批“访惠聚”活动。健全群防群治工作机制，强化驻乌单位维稳责任制，形成全社会人人有责、积极参与、齐抓共管的局面。

三是加强民族团结，促进宗教和谐。全面落实党的民族宗教政策，深入持久开展民族团结宣传教育，让“五个认同”和“三个离不开”思想深入人心。扎实开展民族团结模范社区（村）、模范单位创建活动，开展互学“双语”活动，促进各族群众交流交往交融。持续开展“五好”宗教场所和“五好”宗教人士创建活动，加强爱国宗教人士培训，在宗教界营造爱国感恩、崇尚和谐的良好氛围。

四是扎实推进“去极端化”工作。用好“五把钥匙”，坚持正信挤压、文化对冲、法治约束、科学普及“四管齐下”，加强干部、教师、宗教人士“三支队伍”建设，形成常态化、长效化工作机制。

二、围绕转方式调结构，推动经济提质增效升级

一是抓好“五大任务”落实。按照“去产能、去库存、去杠杆、降成本、补短板”的要求，坚持稳中求进、稳中求变、稳中求好，加快转变经济发展方式，促进产业优化升级并向中高端方向发展。充分运用市场化手段，严格环保、能耗、技术标准，加大钢铁、煤炭、化工、建材等传统产业过剩产能化解力度，加快淘汰落后产能。以房地产去库存为重点，打通供需通道，稳定房地产市场。在继续创新投融资机制、拓宽融资渠道、加大融资力度的同时，积极防范和应对风险。规范政府性债务管理，加强对民间借贷等监管力度，规范融资、担保、贷款等行为，采取有效措施切实降低杠杆率。全面落实国家结构性减税政策，深入清理面向企业的行政收费，精简归并“五险一金”，规范行政审批中介服务，实施低电价、工商业用电同价等政策，打好政策“组合拳”，降低企业生产经营成本，提振企业信心。实施扶企强企行动，通过设立产业引导基金，实行财政贴息、补助等办法，帮助企业渡过难关。加大中小微企业扶持力度，着力解决融资难、融资贵问题。按照“三个引领”的要求，围绕经济社会发展的薄弱环节，着力补上基础设施建设、经济持续健康发展、科技创新进步、城乡统筹发展、民生建设、对外开放、环保生态建设、人才队伍建设等方面的短板。

二是加快推进新型工业化。加快实施汽车、风电、工程机械、电子新材料等优势产业的产业链招商，全力促进龙头企业提质增效，加速提升产业集群效应，引领和带动先进装备制造业基地、高新技术产业基地和战略性新兴产业基地加快发展。推动石油化工、纺织服装、钢铁产业转型升级，大力实施两化融合提升工程，支持企业实施技术改造，全面推行循环生产方式，促进企业、园区、行业间链接共生、原料互供、资源共享。加快智慧城市、天山云产业基地等项目建设。加大重点行业用能监测力度，加快污染企业搬迁、电厂综合治理和小煤矿关闭工作，推进循环化改造示范试点、资源综合利用“双百工程”示范基地项目建设。

三是促进服务业优化升级。把金融作为第一支撑，落实“金融+科技”战略，积极吸引各类金融机构入驻，鼓励金融机构创新金融服务，丰富金融产品，增加有效供给，增强金融服务实体经济能力。加快现代物流、总部经济、电子商务等产业发展，推进多式联运、城市共同配送建设，推动乌鲁木齐国际纺织品服装商贸中心、新疆国际会展中心二期、白鸟湖新区商业街等一批重点项目建设。促进重点领域消费，改造提升传统商贸流通业，积极引进国际国内知名消费品牌，扩大连锁便利规模，推广特色餐饮，增强社区商业服务能力，满足各族群众消费升级需求。加快中国旅游产业创新服务基地建设，建立公共旅游信息平台，启动南山国家旅游度假区创建工作。推进乌鲁木齐县冰雪运动公园、红光山景区欢乐谷等项目规划建设，促进旅游业健康发展。

四是积极推进农业现代化。大力发展节水设施农业，推进设施农业提质增效，改造提升设施农业5000亩，确保蔬菜种植面积稳定在27万亩。推进现代都市农业和休闲观光农业发展，着力打造各类特色农业观光园。扶持建设特种养殖合作社及规范化养殖场10个，创建畜禽水产健康养殖示范区4个。完善农产品质量安全预警和追溯体系，加强生产、仓储和运输环节全过程监管。创新农业经营组织方式，引导农民专业合作社开展信用互助试点。坚持城乡统筹发展，积极推进美丽宜居乡村建设，继续完善农牧区供排水、农田水利等基础设施，建设安居富民住房1万户、农村公路95公里，不断改善农村面貌和农牧民生产生活条件。

三、强化创新驱动发展，营造大众创业、万众创新环境

一是增强自主创新能力。把创新作为第一动力，坚持以推动科技创新为核心，大力开展科技支撑产业行动，推进重大科技专项，鼓励企业提升自主创新能力，促进科技成果转化落地，形成一批拥有自主知识产权的新产品，力争在重要领域和关键环节取得新突破。新增高新技术企业及市级创新型试点企业30家，企业技术中心3家、产学研联合开发示范基地3家、市级工程技术研究中心及重点实验室6家，启动检验检测产业园区建设。

二是加快创新型城市建设。依托丝绸之路经济带创新驱动发展试验区建设，加强对国内外先进技术的引进、消化吸收和再创新，有效促成各类创新资源合作。完善科技资源和科研基础设施开放共享机制，大力推进创新创业基地、众创空间等平台建设，提高创新载体辐射能力和专业服务能力。完善知识产权创造、运用、保护、交易制度建设，专利申请量增长8%，有效发明专利万人拥有量达6件。建立区（县）创新评价机制，增强区（县）创新发展活力。落实完善鼓励创新创业的扶持政策，建立诚信经营、公平竞争的市场环境。加快高新技术产业开发区（新市区）“创新人才培养示范基地”等重点创新园区建设。

三是营造良好创新创业环境。构建有利于大众创业、万众创新的政策制度环境和公共服务体系，激发市场主体活力和创新创业热情。加快推进创新创业载体建设，建立一批小企业创业基地、创业指导服务平台和中小微企业信用服务平台，建立创新创业大赛常态化机制。加强创新创业指导服务，实施高校毕业生创业引领计划，完成创业培训1.1万人。完善人才引进工作机制，实施“创新创业领军人才计划”，加快三个国家级开发区人才试验区建设，加强项目引才，重点吸引科研创新团队、领军人才、高技能人才和海外高层次留学人员来首府创业发展，打造首府创新型人才高地。

四、全面深化改革开放，加快建设丝绸之路经济带核心区“五大中心”

一是深化重点领域改革。按照推进供给侧结构性改革的部署，发挥市场在资源配置中的决定性作用，提高劳动力、资本、技术等全要素生产效率，改善供给质量，优化供给结构，增强发展内生动力。推动简政放权向纵深发展，加快建立完善政府权力、市场准入和政府责任“三个清单”，推进企业信用信息联合监管。深化国有企业改革，以管资本为主加强国有资产监管，完善国有资产管理体制和现代企业制度，分类推进国有企业改革，积极推广政府与社会资本合作（PPP）投融资模式。加快混合所有制经济发展，推进央企属地法人注册及合资合作。优化发展环境，创新体制机制，引导扶持非公有制企业做大做强，增强市场经济活力。推进政府购买公共服务，提高公共服务供给质量。推进财政预算管理改革，重点保障基础设施和民生领域支出。推进农村土地承包权和草原承包权确权登记颁证工作。

二是全方位扩大对外开放。扎实做好第五届中国—亚欧博览会服务保障工作，办好丝绸之路国际食品展交会等12个大型会展活动。积极开展“一带一路”项目投资与合作，吸引重大项目落户，保持引进区外到位资金稳定增长。组织具备条件的二类口岸、大型商品集散地或特色专业市场，积极申报国家内外贸结合商品市场试点。支持境外投资规模化、多元化，推进境外工业园建设。加快电子口岸建设，推动关检合作“三个一”通关模式，提高通关便利化水平。统筹进出口协调发展，扩大深加工特色农产品、高新技术等地产品出口规模，加强市场短缺的能源、资源、原材料和耐用消费品进口，引导鼓励更多外贸企业“走出去”，开展服务外包和跨境电商业务，不断提高城市经济外向度。

三是全力打造丝绸之路经济带核心区“五大中心”。全面落实“五大中心”专项规划，加大区域资源整合力度，夯实“五大中心”基础，增强对中亚及周边地区的辐射力。完善乌昌石交通网络体系，推进高铁新客站基础设施配套工程和临空产业园区建设。加快综合保税区核心区建设，年内实现封关运营，打造创新型新区和兵地融合示范先行先试区。整合现有物流资源和平台，推进乌鲁木齐铁路口岸、多式联运海关监管中心和中欧班列新疆集结中心建设，打造丝绸之路经济带国际物流中心。推进亚欧商贸物流职业教育培训中心、中德合作汽车专业实训基地、丝路国际学校等教育领域国际合作项目。加快乌鲁木齐国际医院、友爱医院建设，积极引进大型医药企业和高端医疗设备企业，完善区域人口健康信息平台和跨境远程医疗信息平台，办好不同主题的丝绸之路健康论坛，探索建立健康产业园，推动医疗旅游服务。

五、提升规划建设管理水平，加快建设现代化国际城市

一是发挥规划引领和调控作用。坚持高起点、高水平、高效益，以主体功能区规划为基础，推动以城市总体规划为龙头的“多规合一”。合理把握规划实施的阶段重点和建设节奏，制定实施好年度发展计划，确保“十三五”经济社会发展规划纲要和117个专项规划得到落实。完善规划编制体系，强化新区规划战略研究，深化完善老城区改造提升规划，优化调整乡镇（村）规划，实现中心城区控制性详细规划全覆盖。注重加强城市设计工作，精心塑造城市景观和宜居环境，不断提升城市品质。加快完善现代化国际城市功能，提高新区教育、医疗、生活服务、文化娱乐等配套水平，加快老城区重点商圈和背街小巷改造提升，创造有吸引力的投资发展环境，引领城市发展全面升级。

二是完善以综合交通枢纽为先导的基础设施建设。加快完善城市路网骨架，实施国道216线提升改造和城北主干道、喀什路、苏州路东延工程，完成新医路西延续建和城北主干道机场高速互通立交工程，实施三屯碑路西延新建工程。加快地铁1号线和2号线一期建设，启动3号线、4号线建设和5号线、6号线前期工作，启动市区至南山中低速磁悬浮和乌昌轻轨规划建设，加快推进国际机场四期改扩建工程。加快750千伏和220千伏输变电项目建设，实施地下综合管廊建设。启动河西污水处理厂、七道湾污水处理厂提标改造工作，关停大浦沟垃圾填埋厂，启用米东垃圾综合处理厂。

三是提升城市精细化管理水平。深入推进环境综合整治，开展城市出入口、城乡结合部等重点区域环境整治，实施“六大公园”和水磨河流域、十七户湿地等景观提升工程，营造干净整洁、清新靓丽的城市环境。加快推进“公交都市”建设，建成BRT6号线支线，优化完善公交线路，配套建设公交场站，开展郊区线路分段计价收费试点工作。进一步完善交通路网，落实公交优先，加强交通管理，多措并举改善城市交通环境。实行公共停车场统一规范管理，新建一批立体停车库，缓解停车难问题。整合优化数字化城市管理平台，建立城市地理信息系统和管线综合动态管理系统，加快数字化城市管理向智慧化升级。强化规划实施管理和国土执法监管力度，坚决遏制违法建设。

四是全面加强生态文明建设。围绕创建国家环保模范城市，继续实施区域大气污染联防联控，严格落实“八个一律”措施，巩固大气污染防治成果。实施1500蒸吨燃气锅炉烟气余热冷凝改造和换热站自动化节能改造项目，推行供热行业差别化定额能耗管理和考核制度。严格执行绿色建筑设计标准，不断提高建筑节能水平。实施既有建筑供热计量及节能改造504万平方米，改造老旧供热管网50公里。继续推进天然气三期输配系统扩建改造、天然气高压环网建设等项目，加快高铁片区和达坂城区电热采暖试点工程。强化机动车尾气治理，淘汰“黄标车”及“老旧车”1万辆。大力开展国家生态园林城市创建工作，新增绿化面积3万亩，森林抚育2万亩。新建荒山绿化公园8个、小游园及街旁绿地100个。围绕构建首府绿色生态屏障，着力推进绕城生态圈建设。积极申办第十二届中国国际园林博览会，启动森林公园申报工作。扎实做好一号冰川保护、柴窝堡生态补湖和湿地保护工程，确保饮用水源和生态系统安全。扩大再生水在城市用水的比例，切实提高水资源利用水平。做好雨水、雪水收集利用工作，推进海绵城市建设。全面实施最严格的耕地保护、水资源管理和环境保护制度，形成资源节约、环境友好的生产和消费方式。推进节能减排财政政策综合示范城市建设。加强环境空间管控和准入管理，完善生态损害赔偿制度和排污许可管理制度，健全生态补偿机制。启动企业环境信用评价工作，加大重点企业排污治理力度。推进矿产资源清洁绿色开发，加强矿山地质环境和煤田火区治理。

六、着力保障和改善民生，增进各族群众福祉

一是促进就业再就业。坚持就业优先战略，创造更多就业岗位，着力解决结构性就业矛盾。实施“千名大学生创业扶持计划”，应届高校毕业生就业率达到85%以上。支持劳动者灵活就业和企业吸纳就业，完善就业困难人员实名制动态管理机制，有序组织农业富余劳动力转移就业。着力提升劳动者职业技能水平，启动免费专项技能培训和高技能人才培养计划，完成职业培训10.5万人。推进和谐劳动关系建设，保障劳动关系双方合法权益。

二是切实增强社会保障能力。加快建立更加公平、更可持续的社会保障制度，统筹城乡公共就业和社会保障服务设施建设，完善城乡居民基本养老、医疗保险制度，推进医疗保险异地就医结算服务，健全工伤保险费率调整和失业动态监测机制。扎实推动社会保险“全民参保计划”，稳步提高社会保险、社会救助等保障水平。提高城乡低保标准，加大扶贫帮困力度，切实保障困难群众的基本生活。

三是推进教育医疗健康事业发展。加快推进教育强区（县）工作，全面提高义务教育发展水平，所有区（县）实现义务教育均衡发展目标。不断优化教育资源配置，推进优质教育资源北扩，加快市第1中学新校区建设，确保市第13中学新校区今年招生办学。加大学前教育投入力度，积极扶持普惠性公益性民办幼儿园发展。创新职业教育办学模式，整合职业教育资源，推动职业教育集团化办学。全面提升“双语”教育水平，保障残疾少年儿童进入普通学校随班就读。完善公共卫生和医疗服务体系，健全区域分级诊疗、重特大疾病保障及疾病应急救助制度，促进基本公共卫生服务均等化，提升医疗服务能力。深化公立医院改革，重点推进县级医院和基层医疗卫生机构综合改革，提高基本药物使用率，破除以药补医机制，减轻群众就医负担。深化食品药品监管体制改革，加强检验检测体系建设，切实保障食品药品安全。全面落实安全生产责任制，建立安全生产预防控制体系，切实提高安全生产治理能力和保障水平，坚决遏制重特大事故发生。广泛开展全民健身活动，推进公共体育设施建设，切实提高各族群众身体素质和健康水平。启动乌鲁木齐奥林匹克体育中心项目，做好第十三届全国冬运会参赛组织和服务保障工作，推进体育产业联系点建设。促进老龄、妇女儿童和红十字事业，推动统计、外事、工商、质监、人防、气象、档案、新闻出版、史志编纂等各项社会事业蓬勃发展。

四是切实改善群众居住条件。完成1.5万户棚户区改造任务，利用存量商品房加快推进安置工作。完善住房保障体系，适度放宽住房保障申请标准，扩大保障范围。在“两房并轨”的基础上，制定公租房分档计租、分级补贴制度。认真落实加强住宅小区物业服务管理的意见，加快推进物业服务市场化、规范化、标准化建设。继续做好生命线工程及公共建筑抗震加固工作。

五是继续开展“民生改善年”活动。确定并落实好今年改善民生100件实事，切实解决好群众反映强烈的突出问题，着力提高公共服务能力，让各族群众有更多参与感、获得感、幸福感。完善社区蔬菜副食品直销点运营管理，确保粮油肉菜供应，保持物价总水平基本稳定。落实残疾人免费乘坐公交车、免费停车及就业扶持补贴等特惠政策。

七、发挥现代文化引领作用，加快建设首善之城

全力推进全国文明城市创建工作，健全创城长效管理机制，争取在薄弱环节和难点问题取得突破，确保顺利通过中央文明委第二年度创城测评验收。把核心价值观宣传教育贯穿于文明城市创建全过程，融入各行各业和群众生产生活，强化正面宣传引导，深入推进“四大文明创建”和文明细胞创建活动。大力开展现代都市文明教育，倡导现代文明理念和行为方式，不断提高市民文明素质和社会文明程度。支持国防和军队改革，推进军地服务业融合发展，巩固全国双拥模范城“八连冠”创建成果。深入推进文化惠民工程，加快构建现代公共文化服务体系，加快市文化中心“六馆”及区（县）图书馆、文化馆等一批重点文化设施建设，推进广播电视“户户通”工程和公益电影放映，不断创新文化讲坛、中老年艺术节等品牌活动。建立全市公共文化服务反馈评估和考核体系。做好“红山文艺奖”评选工作，加快创作一批叫得响、留得住的文艺精品。加大优秀民间文化开发保护传承力度。支持新兴文化业态发展，加快推进文化创意和旅游、体育及相关服务产业融合发展，打造具有地域和民族特色的文化产业品牌。推进特色文化街区、文化产业园区和文化产业示范基地建设，推动骨干文化企业做大做强。积极组织参加深圳文博会等文化产业展会，推动文化企业、特色文化产品“走出去”。

八、进一步加强政府自身建设，不断提高服务能力和水平

进一步坚持党的领导，在思想上、政治上、行动上与中央、自治区党委、市委保持高度一致，这是我们做好政府工作的根本保证，政府的各项工作都要始终在党的领导下进行，要不折不扣地抓好市委各项决策部署的贯彻落实，在这方面，政府要当好主力军，加快建设法治政府、创新政府、廉洁政府和服务政府，不断提高政府服务能力和水平。

一是坚持依法行政，加快建设法治政府。推进法治首府、法治政府、法治社会一体建设，促进首府治理体系和治理能力现代化。启动“七五”普法和依法治理工作，完善普法责任制，大力开展普法宣传教育和群众性法治文化活动，引导全民守法。依法接受人大及其常委会的监督，自觉接受政协的民主监督，主动接受媒体和公众监督。认真履行宪法和法律赋予的职责，按照职能科学、权责法定、执法严明、公开公正、守法诚信的要求，坚持法定职责必须为、法无授权不可为，严格规范公正文明执法，全面抓好依法行政建设法治政府各项任务落实，进一步提高政府工作法治化水平。健全依法维权和化解纠纷机制，将信访纳入法治化轨道，完善多元化纠纷解决机制。

二是坚持务实高效，加快建设创新政府。加快落实全面深化改革各项任务，努力破除与建设创新政府相违背的思想观念、体制机制障碍，进一步提高政府决策的科学化、民主化水平。大力推进简政放权，实行放管结合，推进监管创新，积极探索行之有效的事中事后监管模式。创新思维方式和工作方法，创造性地解决制约首府经济社会发展、政府高效运转以及与群众切身利益密切相关的热点难点问题，身体力行为群众谋福利、谋福祉，让创新政府建设落到实处、取得实效。

三是坚持多措并举，加快建设廉洁政府。认真落实党风廉政建设“两个责任”，完善惩治和预防腐败体系，加强对重点领域、重点环节的监督管理，以“零容忍”态度坚决惩治腐败。巩固党的群众路线教育实践活动和“三严三实”专题教育成果，将严格执行中央八项规定、自治区党委十条规定和市委十项规定作为一项长期重要工作抓紧抓好，坚持不懈纠正“四风”，持续治理“庸懒散拖奢”，不断改进工作作风。加强廉政监察、执法监察、效能监察和审计监督，加大行政问责力度，强化对行政权力的制约和监督，让来源于人民的权力，真正服务于人民。

四是坚持公开透明，加快建设服务政府。深入推进政务公开，加强网络问政，努力打造阳光政府。建成启用区、市两级政务服务中心（公共资源交易中心），按照“应进必进，能进必进”的原则推进部门入驻，创新管理、强化服务，全面提升行政服务效能。强化实干精神，不断增强理解力、落实力、执行力，争做“四强”干部，把人民群众的期待作为工作的动力，全心全意为群众办实事、办好事，真正把中央、自治区和我市的各项惠民政策落到实处，让改革发展成果惠及各族群众。

各位代表，面对各族市民的新期待，我们深感责任重大；站在发展的新起点，我们信心满怀。让我们更加紧密地团结在以习近平同志为总书记的党中央周围，在自治区党委、自治区人民政府和市委的坚强正确领导下，紧紧围绕社会稳定和长治久安总目标，紧抓丝绸之路经济带发展机遇，奋勇拼搏，开拓创新，为加快建设现代化国际城市和全疆团结和谐、繁荣富裕、文明进步、安居乐业的首善之城而努力奋斗
